# Floating Knee Severity Score (FKS): A Novel Multidimensional Prognostic Model for Predicting Functional Outcomes After Floating Knee Injuries

**DOI:** 10.3390/jcm15062109

**Published:** 2026-03-10

**Authors:** Hakan Uslu, Oguzhan Cicek, Bedirhan Sarı, Fırat Seyfettinoğlu, Yüksel Uğur Yaradılmış, Hasan Ulaş Oğur, Evren Karaali, Osman Çiloğlu

**Affiliations:** 1 Department of Orthopaedic and Traumatology, Adana City Training and Research Hospital, Adana 01230, Turkey; oguzhan.cicek21@hotmail.com (O.C.); firatseyf@yahoo.com (F.S.); ugur_yaradilmis@outlook.com (Y.U.Y.); drevrenkaraali@gmail.com (E.K.); osmanciloglu@gmail.com (O.Ç.); 2 Department of Orthopaedic and Traumatology, Adana Ceyhan State Hospital, Adana 01230, Turkey; bedirhan.sarimd@gmail.com

**Keywords:** floating knee, femur fracture, tibia fracture, functional outcome, prognostic score, FKS-score

## Abstract

**Purpose:** Floating knee (FK) injuries are complex high-energy traumas associated with poor functional outcomes. This study aimed to identify independent predictors of functional prognosis and to develop a novel, multidimensional scoring system to predict long-term functional outcomes. **Methods:** A retrospective analysis was performed on 182 adult patients with ipsilateral femur and tibia fractures treated between January 2010 and December 2023. Functional outcomes were assessed using the Karlström–Olerud criteria and dichotomized as excellent–good versus fair–poor. Variables significant in univariate analysis were entered into a multivariate logistic regression model. Independent predictors were used to construct the FKS-score. Predictive performance was evaluated using ROC analysis. **Results:** Of the 182 patients, 103 (56.6%) achieved excellent–good outcomes, while 79 (43.4%) had fair–poor results. Multivariate analysis identified Fraser IIA–B (OR 2.12), Fraser IIC (OR 3.85), Gustilo I–II (OR 1.42), Gustilo IIIA–B (OR 2.61), Gustilo IIIC (OR 3.22), segmental fractures (OR 2.18), extensor mechanism injury (OR 2.04), vascular injury (OR 4.89), intra-articular extension (OR 1.92), and patella fracture (OR 1.76) as independent predictors of poor functional outcome. The FKS-score, ranging from 0 to 15, demonstrated high predictive accuracy (AUC = 0.89). An optimal cut-off value of ≥9 points yielded a sensitivity of 78% and specificity of 85%. **Conclusions:** The FKS-score is the first comprehensive prognostic scoring system specifically developed for floating knee injuries. It provides a reliable, practical tool for early risk stratification and the prediction of long-term functional outcomes, thereby supporting clinical decision-making and patient counseling.

## 1. Introduction

Floating knee (FK) is a complex injury in which the knee joint loses its bony continuity above and below due to simultaneous ipsilateral femur and tibia fractures [1]. With the advancement of technology, the increased frequency of high-energy trauma has increased the incidence of FK, and it has been reported that these patients frequently experience multi-organ injuries in addition to limb injuries [2]. In these fractures, serious systemic complications such as excessive blood loss, infection, and fat embolism can develop in the early stages [3,4]. On the other hand, in the long term, permanent functional impairments such as limited knee range of motion, reduced muscle strength, limb shortening, joint instability, and inability to bear weight may occur [3,5]. Therefore, it is necessary to determine the appropriate treatment approach based on the type of fracture and accompanying soft-tissue damage [6]. Furthermore, it is of great importance to predict the functional prognosis by identifying risk factors in the preoperative period [6].

In fracture cases, the most commonly used radiological classification, based on fracture type, is the Fraser classification (FS) [2]. In addition, various classification methods have been described in the literature and have contributed significantly to determining fracture type and surgical planning [4,7]. However, these methods focus solely on the anatomical pattern and are limited in their ability to predict functional outcomes [4,7]. It has been shown that considering soft tissues and extensor mechanisms, in addition to classical fracture classifications, better reflects prognosis [6]. Preliminary studies have identified a group of prognostic factors, such as the severity of fracture pattern, presence of open fracture, grade, amount of soft-tissue injury, vascular damage, occurrence of segmental fracture, dislocation of the extensor apparatus, and intra-articular involvement, which have an influence on functional outcomes in floating knee injuries [8,9,10]. However, no practical, clinically applicable scoring system is available in the literature.

Therefore, in our study, we aimed to develop a prognostic scoring system to predict long-term functional outcomes by holistically evaluating clinical parameters, including fracture type, presence of accompanying soft-tissue damage, vascular integrity, and joint mechanism.

## 2. Materials and Methods

This study retrospectively reviewed patients who presented to and were followed up at our clinic for floating knee injuries between January 2010 and December 2023. Prior approval was obtained from our hospital’s ethics committee (No: 8/271/2024). The study was conducted in accordance with the Helsinki Declaration. All patients’ file records, surgical notes, and radiological images were evaluated through the hospital information management system. Inclusion criteria were: being 18 years of age or older, having radiologically confirmed ipsilateral femur and tibia fractures, having complete access to initial assessment and follow-up data, and having at least 12 months of follow-up. Patients with pathological fractures, patients under 18 years of age, cases with primary limb amputation at the time of admission, patients with insufficient clinical or imaging data, periprosthetic or refracture cases, and patients who died after admission were excluded.

After excluding patients who met the exclusion criteria, 182 patients were included in the study. Demographic data for patients, including the mechanism of trauma, time to presentation, fracture severity as defined by the Fraser classification, soft-tissue injury severity according to the Gustilo–Anderson classification, type of initial intervention, joint mechanism involvement, and concomitant organ injuries, were recorded. Joint mechanism involvement was evaluated by documenting extensor mechanism injuries, intra-articular fracture extension into the knee joint surface, and associated patellar fractures based on operative reports and radiological findings. Functional outcomes were evaluated according to the Karlström–Olerud criteria [11]. This system divides patients into four categories based on pain level, knee and hip joint range of motion, walking ability, ability to perform daily activities, and radiological findings of union. Excellent and good results indicate satisfactory functional recovery, while fair and poor results indicate clinically inadequate recovery, significant range-of-motion restriction, or permanent impairment in daily living activities [11]. Fracture patterns were classified using the Fraser classification, categorizing injuries according to diaphyseal and intra-articular involvement of the femur and tibia. The Fraser classification categorizes floating knee injuries into four main types: Type I, involving diaphyseal fractures of both the femur and tibia; Type IIA, consisting of a femoral shaft fracture combined with an intra-articular tibial fracture; Type IIB, consisting of a tibial shaft fracture combined with an intra-articular femoral fracture; and Type IIC, in which both fractures extend into the knee joint surface [2]. Open fractures were graded based on the Gustilo–Anderson classification to reflect the severity of soft-tissue damage and contamination. According to the Gustilo–Anderson classification, open fractures are categorized as Type I (clean wounds smaller than 1 cm with minimal soft-tissue damage), Type II (wounds larger than 1 cm without extensive soft-tissue injury), and Type III (high-energy injuries with severe soft-tissue damage or contamination). Type III fractures are further subdivided into Type IIIA, in which adequate soft-tissue coverage is possible; Type IIIB, characterized by extensive soft-tissue loss requiring flap coverage; and Type IIIC, which involves associated arterial injury requiring surgical repair [12]. All vascular injuries other than Gustilo–Anderson type IIIC, defined as a vascular injury causing impaired tissue perfusion and/or vascular symptoms, were recorded separately as an independent variable. Vascular injury was diagnosed based on clinical findings such as absent or diminished peripheral pulses, signs of limb ischemia (pallor, pain, paresthesia, paralysis, and poikilothermia), and findings from imaging studies. Doppler ultrasonography was used as the initial imaging modality, and computed tomography angiography was performed when vascular injury was clinically suspected or when Doppler findings were inconclusive. The primary endpoint of the study was defined as the classification of functional outcomes into two categories, excellent–good and fair–poor, according to the Karlström–Olerud criteria [11].

### 2.1. Sample Size

As this was a retrospective study, the sample size was determined by the number of eligible cases available. Model adequacy was evaluated using events-per-variable considerations and internal validation with bootstrap resampling.

### 2.2. Scoring System

The Floating Knee Severity Score (FKS-score) was developed using variables identified as independent predictors of poor functional outcome in the multivariate logistic regression analysis. Each variable was assigned a weighted point value based on the magnitude of its odds ratio, with higher odds ratios receiving higher point values. Point values were rounded to the nearest integer to maintain clinical simplicity. This rounding strategy represents a deliberate compromise between statistical precision and practical clinical usability of the scoring system. The total FKS score was calculated by summing the individual component scores, yielding a composite score ranging from 0 to 15 points, with higher scores indicating a greater risk of a poor functional outcome.

### 2.3. Statistical Analysis

In statistical analyses, the distribution characteristics of the data were evaluated using the Shapiro–Wilk test. Normally distributed continuous variables were presented with mean and standard deviation, while variables that did not conform to normality were presented with median and interquartile range. Categorical variables were expressed as numbers and percentages. Student’s *t*-test, Mann–Whitney U test, Chi-square test, and Fisher’s exact test were used for comparisons between groups, as appropriate. To identify variables affecting functional outcome, a univariate analysis was first performed, and parameters with *p* < 0.05 were included in a multivariate logistic regression model. In the regression model created using the backward LR method, independent prognostic factors were identified, with odds ratios and 95% confidence intervals reported. Using the variables identified as significant in the regression analysis, the Floating Knee Severity Score (FKS) was developed. The predictive power of the score on functional outcome was evaluated using the ROC curve. The optimal cutoff value was selected using the Youden Index, and sensitivity and specificity were calculated at this cutoff based on the ROC-derived classification. The scoring system was calibrated using the Hosmer-Lemeshow test, and internal validation was performed using bootstrapping. All statistical analyses were performed using R statistical software (version 4.3.1; R Foundation for Statistical Computing, Vienna, Austria).

## 3. Results

A total of 182 patients were included in this study. The most frequent trauma mechanism was a motorcycle accident (*n* = 78, 42.9%). When concomitant injuries were examined, the most common additional pathology was other bone fractures, detected in 56 patients (30.8%). The most frequently preferred method in the surgical treatment of femur fractures was external fixation (*n* = 62, 34.0%). Similarly, external fixation was the most common treatment method for tibia fractures, used in 74 patients (40.7%). The mean healing time was 4.8 ± 1.9 months for the tibia and 4.1 ± 1.7 months for the femur. Delayed healing times were 9.2 ± 3.4 months for the tibia and 8.9 ± 3.1 months for the femur, respectively. Detailed data on the demographic characteristics of the patients, trauma mechanisms, concomitant injuries, applied treatment methods, and healing times are presented in Table 1. When functional outcomes were evaluated according to the Karlström and Olerud criteria, 45 patients (24.7%) had excellent outcomes, 58 patients (31.9%) had good outcomes, 58 patients (31.9%) had fair outcomes, and 21 patients (11.5%) had poor outcomes. Accordingly, the ratio of excellent to good outcomes was 56.6% (*n* = 103), and the ratio of fair to poor outcomes was 43.4% (*n* = 79). In a multivariate logistic regression analysis, more advanced fracture patterns, as defined by the Fraser classification, were identified as strong independent predictors of poor functional outcomes. While the risk was significantly increased in Fraser IIA-B cases (OR 2.12, 95% CI 1.65–2.41; *p* < 0.001), this increase was more pronounced in the Fraser IIC group (OR 3.85, 95% CI 2.01–7.36; *p* < 0.001). According to the Gustilo–Anderson classification, when closed fractures were considered as a reference, the risk of poor functional outcome was significantly increased in Gustilo I–II fractures (OR 1.42, 95% CI 1.12–2.12; *p* = 0.009). This risk was higher in the Gustilo IIIA–B group (OR 2.61, 95% CI 1.51–4.01; *p* = 0.002), and reached its highest level in Gustilo IIIC cases (OR 3.22, 95% CI 1.72–6.04; *p* < 0.001). Among other independent poor prognostic factors, vascular injury emerged as the strongest predictor (OR 4.89, 95% CI 1.09–7.43; *p* = 0.031). This was followed by segmental fracture (OR 2.18; *p* = 0.010), extensor mechanism injury (OR 2.04; *p* = 0.022), intraarticular extension (OR 1.92; *p* = 0.034), and patellar fracture (OR 1.76; *p* = 0.046), respectively. The results of the multivariate logistic regression analysis are summarized in Table 2.

**Figure 1:**
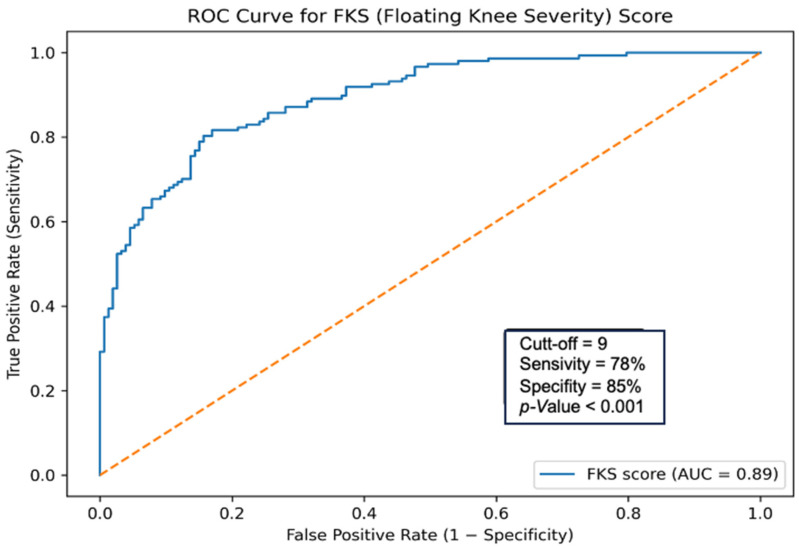
Discriminative performance of the Floating Knee Severity Score (FKS) for predicting poor functional outcomes. The orange dashed line represents the reference line corresponding to a non-discriminative model (AUC = 0.5).

The variables included in the scoring system and their corresponding scores are summarized in Table 3. ROC curve analysis showed that the FKS-score had high predictive performance for poor functional outcomes, with an area under the curve (AUC) of 0.89 (95% CI: 0.83–0.95) (Figure 1). The optimal cutoff value determined using the Youden index was ≥9 points, at which the score’s sensitivity was 78% and its specificity was 85%. The prediction model demonstrated good calibration, as indicated by the Hosmer–Lemeshow goodness-of-fit test (χ^2^ = 6.21, *p* = 0.62). After bootstrap internal validation (1000 resamples), the optimism-corrected area under the curve (AUC) was 0.86 (95% CI: 0.79–0.92), compared with an apparent AUC of 0.89, indicating minimal overfitting.

Early complications were observed in 36 patients (19.7%). The most common early complications were severe bone loss, fat embolism syndrome, and vascular injuries, with a significant proportion of vascular injuries resulting in amputation. Late complications were observed in 31 patients (16.6%); the most frequent were tibial osteomyelitis, knee instability, foot drop, and implant failure. A detailed distribution of early and late complications is presented in Table 4.

## 4. Discussion

Floating knee injuries are complex injuries and can lead to various permanent deformities; predicting the functional prognosis is of great importance [8,9,10]. Our study demonstrated that Fraser Type IIB–IIC fractures, severe open fractures (Gustilo–Anderson II–III), segmental fracture pattern, extensor mechanism injury, and vascular damage were independent negative predictors of functional prognosis. In addition, deterioration in functional outcomes has been observed in cases with compromised joint integrity, such as intra-articular extension and patellar fractures. Based on the current findings, the FKS-score we developed predicts poor functional outcomes with good accuracy (AUC 0.89) and stands out as a reliable, easily applicable model for FI prognosis. In this scoring system, in which each risk factor is assigned an odds ratio, the optimal cut-off was 9 points, with a sensitivity of 78% and a specificity of 85%.

Numerous studies report that Fraser grade, whether the fracture is open or closed, and Gustilo–Anderson grade in open wounds directly affect patient prognosis in fracture injuries [12,13,14,15]. Current large-scale studies have reported that the complication rate is high in Fraser type II injuries and open fracture cases, regardless of the preferred treatment method [13,14]. It has been shown that the good/excellent outcome rates decrease from 74.3% to 33.3% in Fraser Type 1 to Type 2C, and the incidence of infection increases to 10–50% in Gustilo–Anderson Type III open fractures [10,12]. It has been determined that the likelihood of repeated surgery is higher in Grade IIIA–IIIB cases due to the need for debridement caused by infection, and that the Gustilo–Anderson classification is an essential guide for determining the timing and fixation method of surgery [16,17]. In our study, consistent with the literature, we showed that the Fraser pattern of the fracture and the severity of the open injury are independent factors that directly affect prognosis.

In segmental fractures, two separate fracture lines and a free bone segment with impaired blood supply are formed [18]. These fracture types, which are more biomechanically unstable, are considered to have a more difficult healing pattern [18]. While healing is reported to occur in an average of 8.7 months for non-segmental femur fractures, this period is extended to 14.3 months for segmental femur fractures [10]. Due to the more frequent occurrence of complications, such as knee stiffness and limb shortening, associated with significant comminuted and segmental fractures, additional surgical interventions, such as bone grafting, revision fixation, or tendon transfer, are more often needed [9,10,19]. In these cases, factors such as metaphyseal or diaphyseal fracture location, concomitant open fracture, significant displacement, vascular injury, and periosteal dissection have been reported as important risk factors [10]. In our study, when functional outcomes were evaluated according to the Karlström–Olerud criteria, 48.1% of fair-poor cases involved patients with segmental fractures, and this was associated with poor functional outcomes (*p* = 0.0018) [11].

It has been reported that vascular damage occurs in approximately 29% of knee fractures, and the frequency of amputation due to arterial injuries rises to 21% [20]. Popliteal artery and posterior tibial artery injuries are more frequently seen in knee fracture cases, and it has been shown that these vascular injuries have a decisive effect on limb viability and functional outcomes [17,21]. Since Gustilo Type IIIc includes vascular injuries requiring repair, other vascular injuries were also considered as a separate variable in our study and were found to be an important factor affecting prognosis. In these patients, it is recommended to evaluate peripheral pulses during physical examination and to perform a more detailed examination with Doppler ultrasonography if there is clinical suspicion [22]. Furthermore, it is emphasized that, in patients with a foot-arm index < 0.9, the diagnosis should be supported by CT angiography [22].

Extensor mechanism disorders, including patellar fractures and injuries to the patellar or quadriceps tendons, are frequently seen in patellar fracture cases [23]. In a study involving 28 patients, it was reported that in patellar fracture cases, the connection with the extensor apparatus could be severed, and the worst healing rate (3/7) was observed, especially in comminuted patellar fractures [7]. It has also been reported that the coexistence of extensor mechanism injury and intra-articular fracture is a significant risk factor for recurrent surgical procedures and knee stiffness [10]. After the prognostic significance of these parameters was established, the Fraser scoring system was updated and incorporated into the modified Fraser scoring system [7]. In our study, intra-articular fractures were also included.

The preoperative modality of surgical intervention has a provable effect on the functional outcomes after severe trauma to the lower extremities. However, in the present study, the choice of surgical modality depended largely on the nature of the fracture, the presence of soft-tissue injuries, and the overall severity of the injury, rather than being a separate prognostic factor. In turn, the chosen treatment modality virtually reflects the intrinsic injury features and can be viewed as a surrogate measure of injury severity. The addition of surgical modality to the multivariate prognostic model may introduce confounding by indication and collinearity with covariates related to fractures. In this regard, the surgical treatment modality was not included in the final FKS scoring model, which was aimed at generalizing injury-specific prognostic factors obtainable during initial assessment.

## 5. Limitations

One of the primary limitations of this study is its retrospective design and single-center setting, which may limit the generalizability of its findings to broader, more diverse patient populations. In addition, the lack of advanced imaging in all patients may have restricted the detection of certain vascular and soft-tissue injuries. Although the Karlström–Olerud scale has been widely used in previous studies on floating knee injuries, it includes subjective components, and the use of alternative functional outcome assessment tools could have yielded partially different results. Furthermore, although the FKS score demonstrated good discriminative performance in internal validation via bootstrapping, external validation in an independent cohort was not performed. Consequently, the model’s predictive accuracy may be attenuated when applied to external populations with different patient characteristics and trauma care systems, underscoring the need for future multicenter validation studies.

## 6. Conclusions

Our study presents the first scoring system to predict the prognosis of floating-knee impairment cases. We identified independent factors affecting functional outcomes and developed the FKS score using these variables. This scoring system is applicable and can serve as a potential tool for clinical decision-making, with good sensitivity and specificity. This may play an essential role in early risk stratification, correct treatment selection, pre-treatment patient education, and improvement of long-term functional outcomes. However, to more clearly establish the model’s clinical reliability and generalizability, external validation in multicenter, prospective studies is warranted.

## Figures and Tables

**Table 1:** Comparison of Variables Between Outcome Groups.

Variable	Excellent–Good (n = 103)	Fair–Poor (n = 79)	*p*-Value
Age (mean ± SD)	33.7 ± 11.5	33.4 ± 11.0	0.862
Female, n (%)	16 (15.1)	4 (5.3)	0.072
Intra-articular ext., n (%)	23 (22.3)	37 (46.8)	0.0060
Segmental fractures, n (%)	27 (26.2)	38 (48.1)	0.0018
Patella fracture, n (%)	14 (13.6)	22 (27.8)	0.019
Extensor mech. Injury, n (%)	12 (11.6)	23 (29.3)	0.0045
Vascular injury, n (%)	3 (2.8)	8 (10.5)	0.0380
Gustilo class., n (%)			<0.001
Closed	62 (60.2)	13 (16.5)	
Type I	23 (22.3)	20 (25.3)	
Type II	10 (9.7)	16 (20.3)	
Type IIIA	5 (4.9)	11 (13.9)	
Type IIIB	2 (1.9)	11 (13.9)	
Type IIIC	1 (1.0)	8 (10.1)	
Fraser class., n (%)			<0.001
Type I	46 (44.7)	7 (8.9)	
Type IIA	36 (35.0)	29 (36.7)	
Type IIB	16 (15.5)	29 (36.7)	
Type IIC	5 (4.9)	14 (17.7)	

Abbreviations: SD, standard deviation; ext., extension; mech., mechanism; class., classification.

**Table 2:** Multivariate logistic regression analysis for predicting floating knee functional results.

Predictor	OR	95% CI	*p*-Value
Fraser classification (Reference: Type I)	-	-	-
Type IIA–B	2.12	1.65–2.41	<0.001
Type IIC	3.85	2.01–7.36	<0.001
Gustilo classification (Reference: Closed)	-	-	-
Type I–II	1.42	1.12–2.12	0.009
Type IIIA–B	2.61	1.51–4.01	0.002
Type IIIC	3.22	1.72–6.04	<0.001
Segmental fracture	2.18	1.20–3.95	0.010
Extensor mech. Injury	2.04	1.11–3.75	0.022
Vascular injury	4.89	1.09–7.43	0.031
Intra-articular ext.	1.92	1.05–3.52	0.034
Patella fracture	1.76	1.01–3.05	0.046
Age	1.01	0.98–1.03	0.641
Female sex	0.61	0.21–1.67	0.342

Abbreviations: OR, odds ratio; CI, confidence interval; ext., extension; mech., mechanism.

**Table 3:** Floating Knee Severity score (FKS-score) components and point allocation.

FKS Component	Definition	Points
Vascular injury	Vascular injury with impaired tissue perfusion	4
Fraser IIC	Intra-articular fractures of both femur and tibia	3
Gustilo IIIC	Arterial injury requiring surgical repair	3
Gustilo IIIA–B	Severe open fracture	2
Segmental fracture	Segmental femur or tibia fracture	2
Fraser IIA–B	Complex femur–tibia fracture pattern	2
Gustilo I–II	Mild to moderate open fracture	1
Extensor mech. injury	Disruption of quadriceps or patellar tendon	1
Intra-articular ext.	Extension into the knee joint surface	1
Patella fracture	Concomitant patellar fracture	1
Total FKS-score		0–15

Abbreviations: FKS, Floating Knee Score; ext., extension; mech., mechanism.

**Table 4:** Early and Late Complications in 182 Floating-Knee Patients.

Early Complications	n (%)	Late Complications	n (%)
Severe bone loss	9 (4.9)	Osteomyelitis of tibia	8 (4.4)
Fat embolism syndrome	6 (3.3)	Knee instability	6 (3.3)
Vascular injury (limb salvage)	5 (2.7)	Foot drop	5 (2.7)
Vascular injury + primary amputation	5 (2.7)	Implant failure	4 (2.2)
ARDS	4 (2.2)	Osteomyelitis of femur	3 (1.6)
Acute renal failure	3 (1.6)	AVN of femoral head	2 (1.1)
Primary amputation (crush injury)	2 (1.1)	Malunited patella	2 (1.1)
Others	2 (1.1)	Late amputation	1 (0.5)
Total	36 (19.7)	Total	31 (16.6)

## Data Availability

The datasets generated and/or analyzed during the current study are available from the corresponding author upon reasonable request.
